# Characterization of somatic mutation-associated microenvironment signatures in acute myeloid leukemia patients based on TCGA analysis

**DOI:** 10.1038/s41598-020-76048-8

**Published:** 2020-11-04

**Authors:** Jun Wang, Feng-Ting Dao, Lu Yang, Ya-Zhen Qin

**Affiliations:** Peking University People’s Hospital, Peking University Institute of Hematology, National Clinical Research Center for Hematologic Disease, No. 11 Xizhimen South Street, Xicheng District, Beijing, 100044 China

**Keywords:** Cancer microenvironment, Tumour biomarkers, Tumour immunology

## Abstract

Recurrent genetic mutations occur in acute myeloid leukemia (AML) and have been incorporated into risk stratification to predict the prognoses of AML patients. The bone marrow microenvironment plays a critical role in the development and progression of AML. However, the characteristics of the genetic mutation-associated microenvironment have not been comprehensively identified to date. In this study, we obtained the gene expression profiles of 173 AML patients from The Cancer Genome Atlas (TCGA) database and calculated their immune and stromal scores by applying the ESTIMATE algorithm. Immune scores were significantly associated with OS and cytogenetic risk. Next, we categorized the intermediate and poor cytogenetic risk patients into individual-mutation and wild-type groups according to RUNX1, ASXL1, TP53, FLT3-ITD, NPM1 and biallelic CEBPA mutation status. The relationships between the immune microenvironment and each genetic mutation were investigated by identifying differentially expressed genes (DEGs) and conducting functional enrichment analyses of them. Significant immune- and stromal-relevant DEGs associated with each mutation were identified, and most of the DEGs (from the FLT3-ITD, NPM1 and biallelic CEBPA mutation groups) were validated in the GSE14468 cohort downloaded from the Gene Expression Omnibus (GEO) database. In summary, we identified key immune- and stromal-relevant gene signatures associated with genetic mutations in AML, which may provide new biomarkers for risk stratification and personalized immunotherapy.

## Introduction

Acute myeloid leukemia (AML) is a hematological malignancy characterized by the clonal expansion of myeloid blasts, resulting in impaired hematopoiesis and bone marrow failure^1,2^. The outcomes of AML patients are highly heterogeneous, and analysis of cytogenetic abnormalities is the backbone of risk stratification in AML^3^. In recent years, several somatic mutations, such as FLT3 and NPM1, were shown to be strongly prognostic and have been incorporated into risk categories of AML in both NCCN guidelines and ELN recommendations^2,3^.

Tumor microenvironment (TME) infrastructure, which comprises a variety of immune and stromal cell types (e.g., endothelial cells and fibroblasts) and extracellular components they secrete (e.g., cytokines, growth factors, hormones, and extracellular matrix), represents a chronic inflammatory, immunosuppressive, and proangiogenic intratumoral environment^4–7^. The TME not only plays an important role during tumor initiation, progression, and metastasis but also has profound implications for therapeutic efficacy and specificity^8–13^. AML myeloid blasts are able to adapt and grow in bone marrow environments with a significantly lower likelihood of detection and eradication by host immunosurveillance compared with other environments. Recent evidence has highlighted the importance of the bone marrow microenvironment in protecting leukemic stem cells (LSCs) from chemotherapy-induced cell death^14^. Therefore, efforts to characterize the TME signatures have drawn considerable attention in the field of solid tumors, as well as leukemia.

Estimation of STromal and Immune cells in Malignant Tumors using Expression data’ (ESTIMATE) is a method that uses gene expression signatures to infer the fraction of stromal and immune cells in tumor samples^15^. This algorithm has been employed to investigate the microenvironment of several solid tumors, such as gastric cancer^16^, breast cancer^17^ and glioblastoma^18^, and it has also been applied to estimate immune and stromal scores in AML patients^19–21^. Since gene mutations are important prognostic factors for AML, whether they individually have unique microenvironment features has not been determined to date.

In the current study, by downloading gene expression profiles for AML cohorts from The Cancer Genome Atlas (TCGA) database and analyzing the immune/stromal scores of patients based on the ESTIMATE algorithm, we characterized the gene mutation-associated microenvironment. Moreover, the identified immune- and stromal-relevant DEGs associated with some mutations were verified using the Gene Expression Omnibus (GEO) database.

## Results

### OS and impact of immune and stromal scores in AML patients

**Figure 1:**
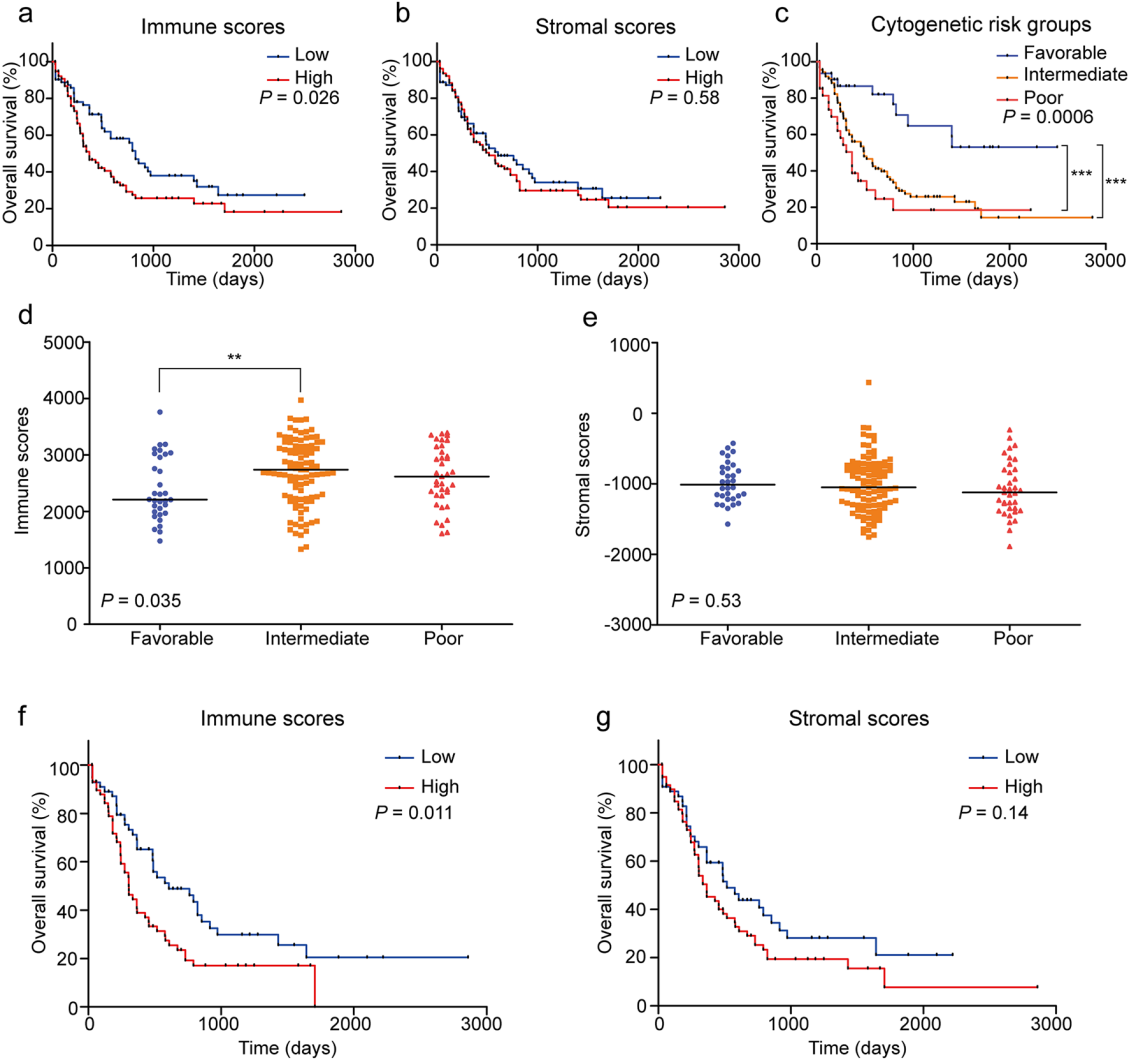
Immune scores and stromal scores are associated with AML OS and cytogenetic risk. (**a**) Kaplan–Meier survival analysis of high versus low immune score groups (log-rank test, *P* = 0.026). (**b**) Kaplan–Meier survival analysis of high versus low stromal score groups (log-rank test, *P* = 0.58). (**c**) Kaplan–Meier survival analysis of cytogenetic risk groups (log-rank test, *P* = 0.0006). (**d**) Distribution of immune scores within cytogenetic risk groups (*P* = 0.035). (**e**) Distribution of stromal scores within cytogenetic risk groups (*P* = 0.53). (**f**) Kaplan–Meier survival analysis of high versus low immune score groups in the intermediate and poor cytogenetic risk patients (log-rank test, *P* = 0.011). (**g**) Kaplan–Meier survival analysis of high versus low stromal score groups in the intermediate and poor cytogenetic risk patients (log-rank test, *P* = 0.14).

The complete gene expression profiles and clinical information of 173 AML patients were retrieved from the TCGA database for this study. The median follow-up period was 304 days (range, 28–2861 days) for the entire cohort, and the 2-year OS rate was 44.4% (95% confidence interval (CI), 35.6–52.8%). According to the ESTIMATE algorithm, immune scores ranged from 1329.53 to 3971.97, whereas stromal scores varied from -1888.81 to 435.75. Then, patients were divided into high- and low-score groups according to the median immune and stromal scores, respectively. Patients with high immune scores had significantly lower 2-year OS rates than did those with low immune scores (32.7% [95% CI 21.9–43.8%] vs 58.1% [95% CI 44.6–69.5%], *P* = 0.026, log-rank test, Fig. 1a). However, patients in the high stromal score group had similar 2-year OS rates to those in the low stromal score group (41.4% [95% CI 29.9–52.4%] vs 48.6% [95% CI 35.1–60.9%], *P* = 0.58, log-rank test, Fig. 1b).

### Relationship between immune/stromal scores and cytogenetic risk

Of the 173 patients, 32 (18.5%) were in the favorable cytogenetic risk group, 101 (58.4%) were in the intermediate cytogenetic risk group, 37 (21.4%) were in the poor cytogenetic risk group, and the remaining 3 (1.7%) belonged to the unknown group. As shown in Fig. 1c, patients in the intermediate cytogenetic risk group had a similar 2-year OS rate as those in the poor cytogenetic risk group (39.7% [95% CI 28.8–50.4%] vs 24.6% [95% CI 9.7–43.0%], *P* = 0.16, log-rank test), and they both had significantly lower 2-year OS rates compared with the favorable cytogenetic risk group (82.0% [95% CI 61.7–92.2%], *P* = 0.0009 and 0.0002, log-rank test).

The median immune scores in the favorable, intermediate and poor cytogenetic risk groups were 2209.75, 2738.40 and 2616.32, respectively (*P* = 0.035, ANOVA test). As shown in Fig. 1d, the immune score of the favorable cytogenetic risk group was significantly lower than those of the intermediate and poor cytogenetic risk groups (*P* = 0.0084 and 0.067, Mann–Whitney test, two-sided), and the immune score of the intermediate risk group was similar to that of the poor risk group (*P* = 0.44, Mann–Whitney test, two-sided). The median stromal scores in the favorable, intermediate and poor risk groups (− 1011.98, − 1050.87 and − 1122.03) were similar (*P* = 0.53, ANOVA test, Fig. 1e).

Overall, patients in the favorable cytogenetic risk group had significantly lower immune scores and a higher 2-year OS rate. However, there were no significant differences in the 2-year OS rate, immune scores or stromal scores between the intermediate and poor cytogenetic risk groups. Therefore, the intermediate and poor cytogenetic risk groups (n = 137) were grouped together for the subsequent analysis. After grouping AML patients with intermediate and poor cytogenetic risk by the median score, those with high immune scores still had significantly lower 2-year OS rates than those with the low immune scores (23.5% [95% CI 13.2–35.5%] vs 48.8% [95% CI 34.0–62.1%], *P* = 0.011, log-rank test, Fig. 1f). Furthermore, patients in the high stromal score group tended to have lower 2-year OS rates compared with patients in the low stromal score group (29.1% [95% CI 18.0–41.1%] vs 43.8% [95% CI 28.9–57.8%], *P* = 0.14, log-rank test, Fig. 1g).

### Somatic mutation-associated immune/stromal scores in the intermediate and poor cytogenetic risk groups

**Figure 2:**
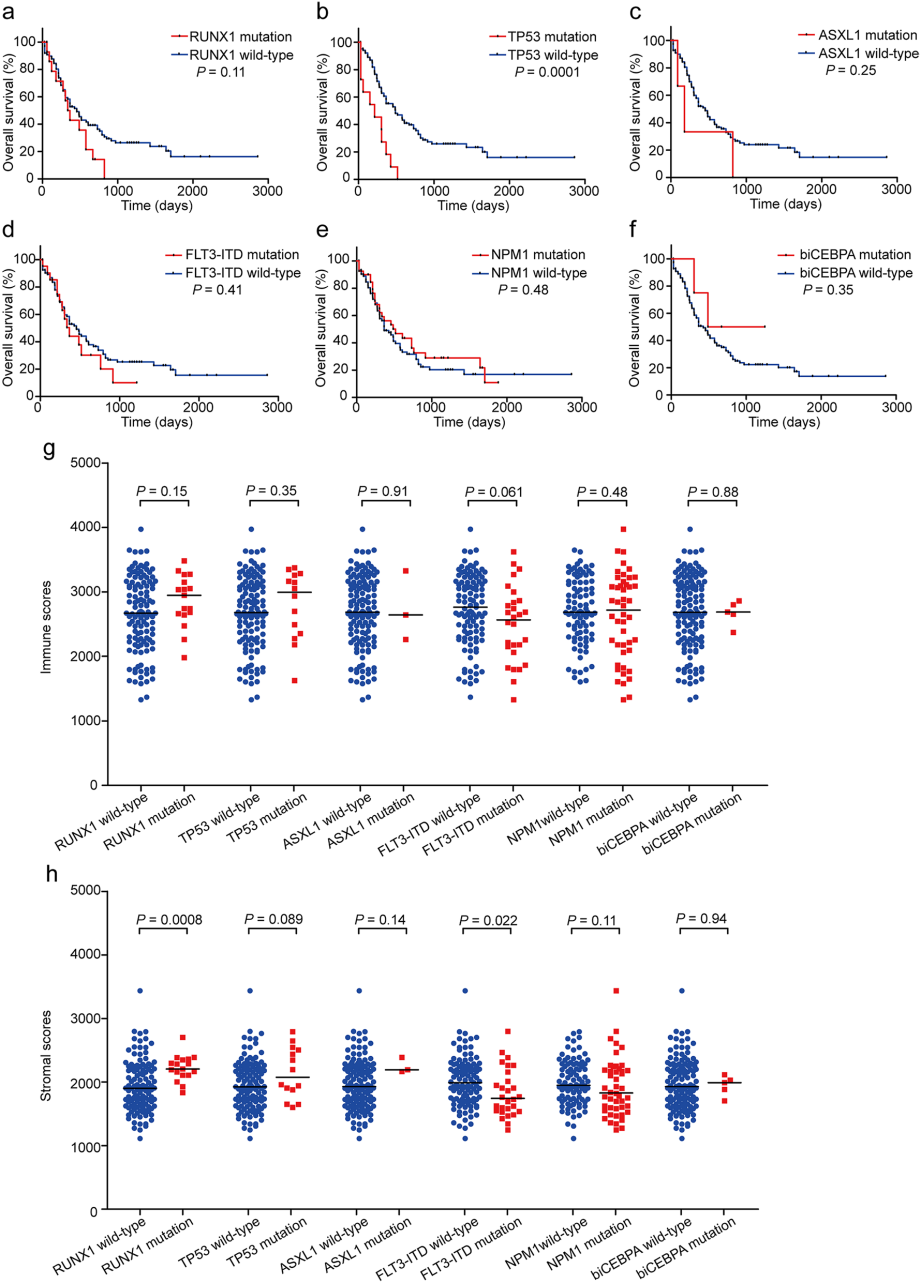
Mutations associated immune and stromal scores in the intermediate and poor cytogenetic risk groups. (**a**–**f**) Kaplan–Meier survival analysis of genetic mutation versus WT groups. (**g**,**h**) Immune and stromal scores of the genetic mutation versus WT groups.

To further explore the association between immune/stromal scores and mutations, patients in the intermediate and poor cytogenetic risk groups were divided into two subgroups based on whether the individual somatic mutation existed, and their immune and stromal scores are presented in Fig. 2g,h.

#### Poor prognostic mutations (RUNX1, TP53, ASXL1 and FLT3-ITD)

As shown in Fig. 2a–d, patients with the individual gene mutation had or tended to have lower 2-year OS rates than WT patients (RUNX1: 14.3% [95% CI 2.3–36.6%] vs 39.2% [95% CI 28.9–49.2%], *P* = 0.11, Log-rank test; TP53: 0% [95% CI 0–0%] vs 39.7% [95% CI 29.6–49.6%], *P* = 0.0001, Log-rank test; ASXL1: 33.3% [95% CI 0.9–77.4%] vs 35.5% [95% CI 26.1–45.0%], *P* = 0.25, Log-rank test; FLT3-ITD: 30.2% [95% CI 10.5–52.9%] vs 36.6% [95% CI 26.4–46.8%], *P* = 0.41, Log-rank test), respectively.

Patients in the RUNX1 and TP53 mutation groups had or tended to have higher immune scores and stromal scores than did those in the corresponding WT groups (RUNX1: immune score 2947.2 vs 2669.7, *P* = 0.15, Mann–Whitney test, two-sided; stromal score − 794.34 vs − 1098.615, *P* = 0.0008, Mann–Whitney test, two-sided. TP53: immune score 2993.49 vs 2670.13, *P* = 0.35, Mann–Whitney test, two-sided; stromal score − 923.14 vs − 1073.74, *P* = 0.089, Mann–Whitney test, two-sided). ASXL1 mutation patients had similar immune scores compared with WT patients (2642.07 vs 2685.88, *P* = 0.91, Mann–Whitney test, two-sided), but they tended to have higher stromal scores than WT patients (− 806.38 vs − 1072.71, *P* = 0.14, Mann–Whitney test, two-sided). However, both the immune scores and stromal scores of patients with FLT3-ITD mutation were lower or tended to be lower than those of WT patients (immune score 2565.07 vs 2762.73, *P* = 0.061, Mann–Whitney test, two-sided; stromal score -1255.38 vs -1010.38, *P* = 0.022, Mann–Whitney test, two-sided) (Fig. 2g,h).

#### Favorable prognostic mutations (NPM1 and biCEBPA)

Patients in the NPM1/biCEBPA mutation groups individually tended to have higher 2-year OS rates than those in the WT groups (NPM1: 43.3% [95% CI 26.6–59.1%] vs 31.7% [95% CI 20.9–42.9%], *P* = 0.48, Log-rank test, Fig. 2e; biCEBPA: 50.0% [95% CI 5.8–84.5%] vs 35.0% [95% CI 25.6–44.5%], *P* = 0.35, Log-rank test, Fig. 2f).

NPM1 mutation patients tended to have lower stromal scores than WT patients (− 1172.08 vs − 1050.87, *P* = 0.11, Mann–Whitney test, two-sided), but their immune scores were similar (2716.05 vs 2683.65, *P* = 0.48, Mann–Whitney test, two-sided). Patients with biCEBPA mutation had similar immune scores and stromal scores compared with WT patients (immune score 2688.10 vs 2683.34, *P* = 0.88, Mann–Whitney test, two-sided; stromal score − 1010.38 vs − 1070.96, *P* = 0.94, Mann–Whitney test, two-sided) (Fig. 2g,h).

The above analysis reflected that there were distinct relationships between somatic mutations and immune/stromal scores. RUNX1 and TP53 mutations were related to both higher immune scores and higher stromal scores, FLT3-ITD was related to both lower immune scores and lower stromal scores, ASXL1 mutation was only related to higher stromal scores, NPM1 was only related to lower stromal scores, and biCEBPA was not related to immune scores and stromal scores.

### Identification of differentially expressed genes (DEGs) and functional enrichment analysis

As shown in Supplementary Figure S1, after the gene expression data of patients with intermediate and poor cytogenetic risk was analyzed, a certain number of upregulated and downregulated genes were identified for the individual somatic mutations RUNX1, TP53, ASXL1, FLT3-ITD, NPM1 and biCEBPA.

**Figure 3:**
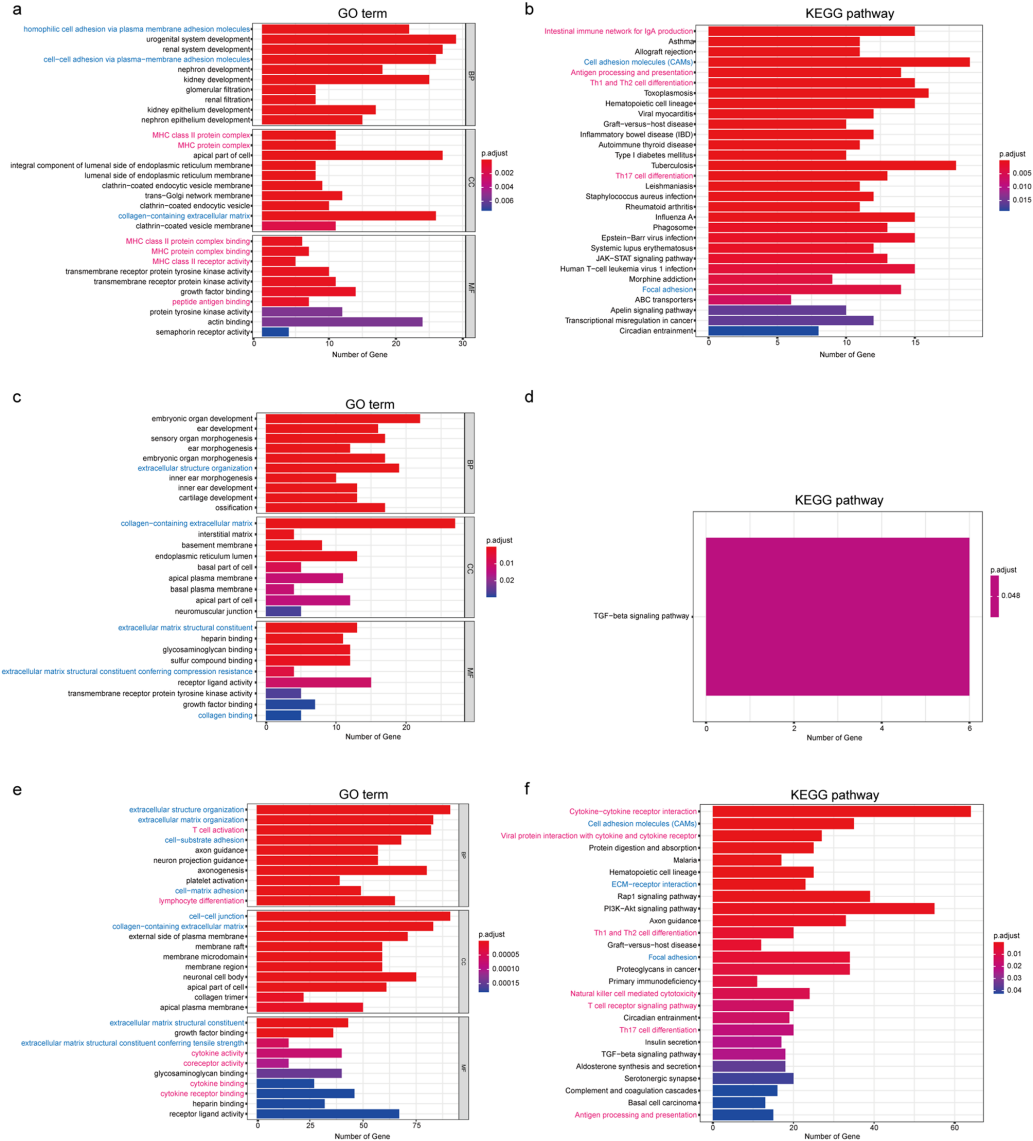

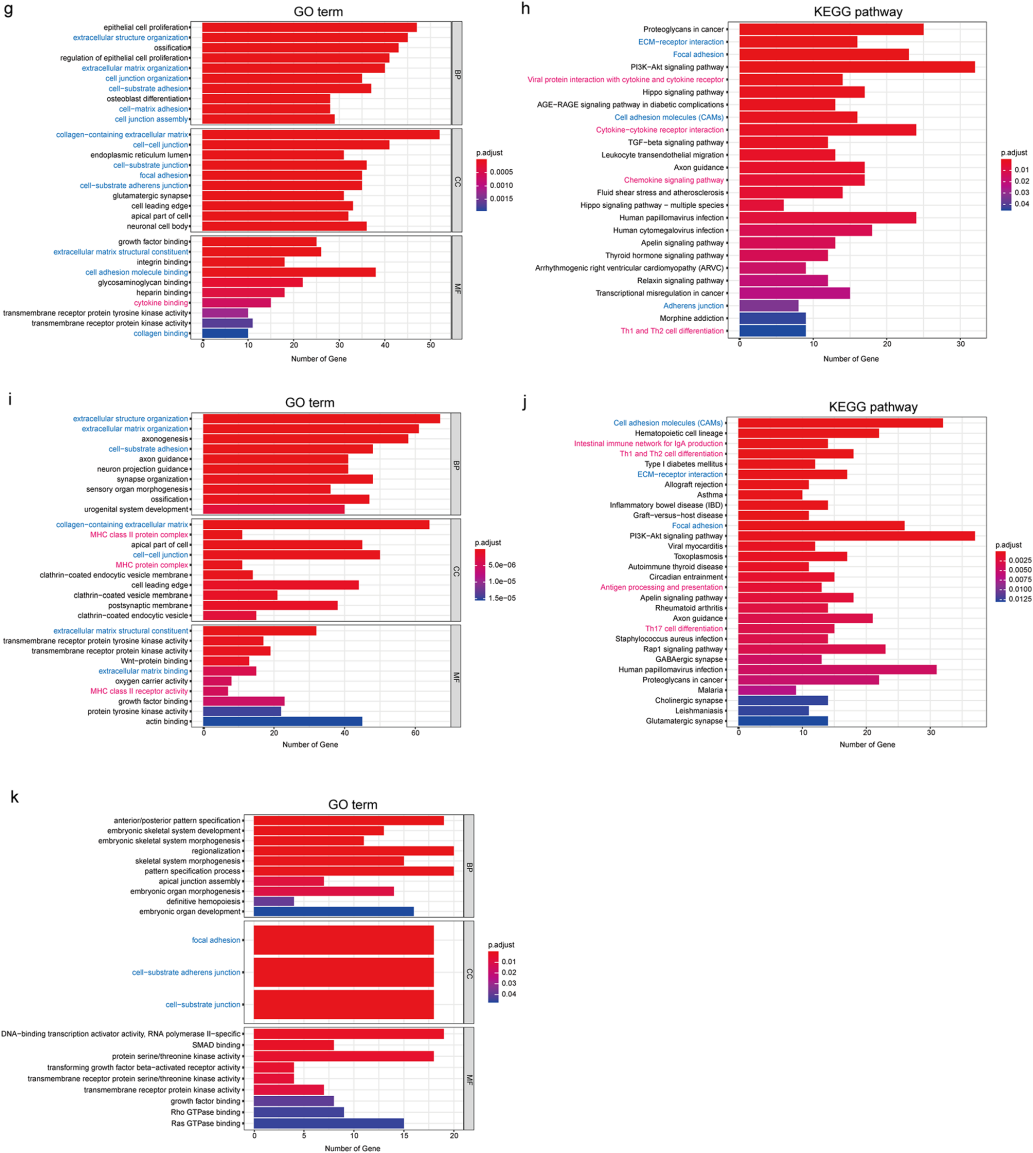
GO term and KEGG pathway enrichment analysis of DEGs. (**a**,**b**) GO terms and KEGG pathways of RUNX1 upregulated genes. (**c**,**d**) GO terms and KEGG pathways of ASXL1 upregulated genes. (**e**,**f**) GO terms and KEGG pathways of TP53 upregulated genes. (**g**,**h**) GO terms and KEGG pathways of FLT3-ITD downregulated genes. (**i**,**j**) GO terms and KEGG pathways of NPM1 downregulated genes. (**k**) GO terms of biCEBPA downregulated genes. Note: immune-related pathways are marked *red,* and stromal-related pathways are marked *blue*.

The results of GO term and KEGG pathway enrichment analysis are shown in Fig. 3. In general, the enrichment analysis results were consistent with the immune/stromal scores. In other words, both immune- and stromal-related GO terms and KEGG pathways were enriched for RUNX1, TP53 and FLT3-ITD mutations, which corresponded to their association with immune and stromal scores. Only stromal-related GO terms were enriched for ASXL1 mutation, which corresponded to its only association with stromal scores. However, discrepancies were observed for NPM1 and biCEBPA mutations: both immune- and stromal-related GO terms and KEGG pathways were enriched for NPM1 mutation, although it was only related to lower stromal scores; stromal-related GO terms were enriched for biCEBPA mutation, despite its lack of a relationship to immune/stromal scores.

### Characteristics and prognostic significance of mutation-associated immune/stromal cell-relevant DEGs

Mutation-associated immune/stromal score-relevant DEGs were selected according to ESTIMATE algorithm gene lists and are shown in Supplementary Table S1. Overall, the number of immune/stromal cell-relevant DEGs was consistent with immune/stromal scores (Table 1): for RUNX1 and TP53 mutations, the majority of both immune and stromal cell-relevant DEGs were upregulated, which corresponded to their associated higher immune and stromal scores; for the ASXL1 mutation, only several stromal cell-relevant DEGs were upregulated, which corresponded to its associated higher stromal scores; the majority of both immune and stromal cell-relevant DEGs for FLT3-ITD and the majority of stromal cell-relevant DEGs for NPM1 mutation were downregulated, which corresponded to their associated lower scores; for biCEBPA mutation, almost no immune and stromal cell-relevant DEGs were observed, which corresponded to its lack of a relationship with immune and stromal scores. The only exception was that the majority of immune cell-relevant DEGs for NPM1 mutations were downregulated, despite their lack of association with immune scores.

**Table 1:** Characteristics of somatic mutation-associated immune/stromal cell-relevant DEGs.

Gene	Immune		Stroma	
	Score (*P* values)	Number of DEGs* (Mu vs WT)	Score (*P* values)	Number of DEGs* (Mu vs WT)
RUNX1	Mu > WT (0.15)	Up: 8 down: 0	Mu > WT (0.0008)	Up: 17 down: 2
TP53	Mu > WT (0.35)	Up: 24 down: 2	Mu > WT (0.089)	UP: 41 down: 0
ASXL1	Mu ≈ WT (0.91)	Up: 0 down: 0	Mu > WT (0.014)	Up: 7 down: 1
FLT3-ITD	Mu < WT (0.061)	Up: 0 down: 7	Mu < WT (0.022)	Up: 2 down: 20
NPM1	Mu ≈ WT (0.48)	Up: 2 down: 11	Mu < WT (0.11)	Up: 5 down: 27
biCEBPA	Mu ≈ WT (0.88)	Up: 1 down: 2	Mu ≈ WT (0.94)	Up: 1 down: 1

*Only these involved in the ESTIMATE algorithm gene list were considered.

There were overlaps among the individual mutation-associated immune/stromal cell-relevant DEGs (Supplementary Fig. S2). The common upregulated stromal cell-relevant DEGs among RUNX1, TP53 and ASXL1 mutations included DDR2 and FRZB. The common downregulated immune cell-relevant DEGs between FLT3-ITD and NPM1 mutations included CD3D, CD48, GBP1, and IL18RAP. The common downregulated stromal cell-relevant DEGs between FLT3-ITD and NPM1 mutations included BGN, CDH5, COL1A2, COL6A3, CXCL12, DCN, FRZB, ISLR, ITIH5, MXRA5, TRAT1, and VCAM1 (n = 12). Furthermore, FRZB was the only DEG associated with all 5 mutations (RUNX1, TP53, ASXL1, FLT3-ITD and NPM1), ITIH5 was associated with RUNX1, ASXL1, FLT3-ITD and NPM1 mutations, and ISLR was associated with TP53, ASXL1, FLT3-ITD and NPM1 mutations.

The intermediate and poor cytogenetic risk patients were grouped by the median transcription levels of the individual mutation-associated immune/stromal cell-relevant DEGs to evaluate their effects on OS. The following genes were found to have prognostic significance, which was consistent with that of the corresponding mutation: high expression of SCUBE2 (RUNX1 mutation-associated upregulation) was shown to be related to lower OS, high expression of SPON1 (TP53 mutation-associated upregulation) was shown to be related to lower OS, high expression of GREM1 (ASXL1 mutation-associated upregulation) was shown to be related to lower OS, low expression of COL3A1, CXCL12, EMCN, FRZB, ITIH5, KDR, MXRA5, TART1, and VCAM1 (FLT3-ITD mutation-associated downregulation) were shown to be related to lower OS, high expression of ADAMTS5 (NPM1 mutation-associated upregulation) was shown to be related to higher OS, and low expression of SPON1 (NPM1 mutation-associated downregulation) was shown to be related to higher OS (Supplementary Fig. S3).

### Validation in the GEO database and identification of hub genes

To verify whether these immune and stromal cell-relevant DEGs identified from TCGA AML patients are also associated with mutations in an independent AML cohort, we analyzed the gene expression levels of 524 AML cases from GSE14468, for which FLT3-ITD, NPM1 and biCEBPA mutation data were available. For immune score-involved genes, 7/7 of FLT3-ITD-associated, 13/13 of NPM1 mutation-associated and 3/3 biCEBPA mutation-associated DEGs were confirmed to be significantly related to the individual gene mutation by GSE14468. Similarly, for stromal score-involved genes, 7/22 FLT3-ITD-associated, 20/32 NPM1 mutation-associated and 2/2 biCEBPA mutation-associated DEGs were also confirmed. The confirmed mutation-associated immune and stromal cell-relevant DEGs are shown in Table 2.

**Table 2:** FLT3-ITD, NPM1 and biCEBPA mutations-associated immune and stromal cell-relevant DEGs.

Mutation	Status of DEGs	Immune cell-relevant DEGs	Stromal cell-relevant DEGs
FLT3-ITD mutation	Upregulation	None	*ENPP2*; *PTGIS* (n = 2)
	Downregulation	*CD3D*; *CD48*; *GBP1*; *IL10RA*; *IL18RAP*; *IL2RB*; *LCK* (n = 7)	AOC3; BGN; C1QB; CDH5; COL15A1; COL1A2; COL3A1*; *COL6A3*; CXCL12*; *DCN*; EMCN*; FRZB*; ISLR; ITIH5*; KDR*; MXRA5*; PDE2A; RARRES2; TRAT1*; VCAM1* (n = 20)
NPM1 mutation	Upregulation	*CSTA*; *SRGN* (n = 2)	ADAMTS5*; *C3AR1*; *F13A1*; *TNN*; *RASGRP3* (n = 5)
	Downregulation	*CD3D*; *CD48*; *GBP1*; *GZMB*; *HLA-DPA1*; *HLA-DPB1*; *IRF8*; *HLA-DRA*; *IL18RAP*; *RGS1*; *ZAP70* (n = 11)	BGN; *CD200*; *CD248*; CDH5; COL1A2; *COL6A3*; *COL8A2*; *CXCL12*; DCN; *EDIL3*; ENPEP; *ERG*; *FBLN2*; FRZB; *HGF*; *IL18R1*; ISLR; ITIH5; *ITM2A*; *MXRA5*; PAPPA; PRKG1; SIGLEC1; SPON1*; SULF1; *TRAT1*; *VCAM1* (n = 27)
biCEBPA mutation	Upregulation	*IL2RG* (n = 1)	*ITM2A* (n = 1)
	Downregulation	*GBP2*; *IL4R* (n = 2)	*F13A1* (n = 1)

Genes that displayed in *italics* were confirmed to be associated with mutations by GSE14468 data. *These mutation-associated genes have prognostic significance in the intermediate and poor cytogenetic risk patients.

**Figure 4:**
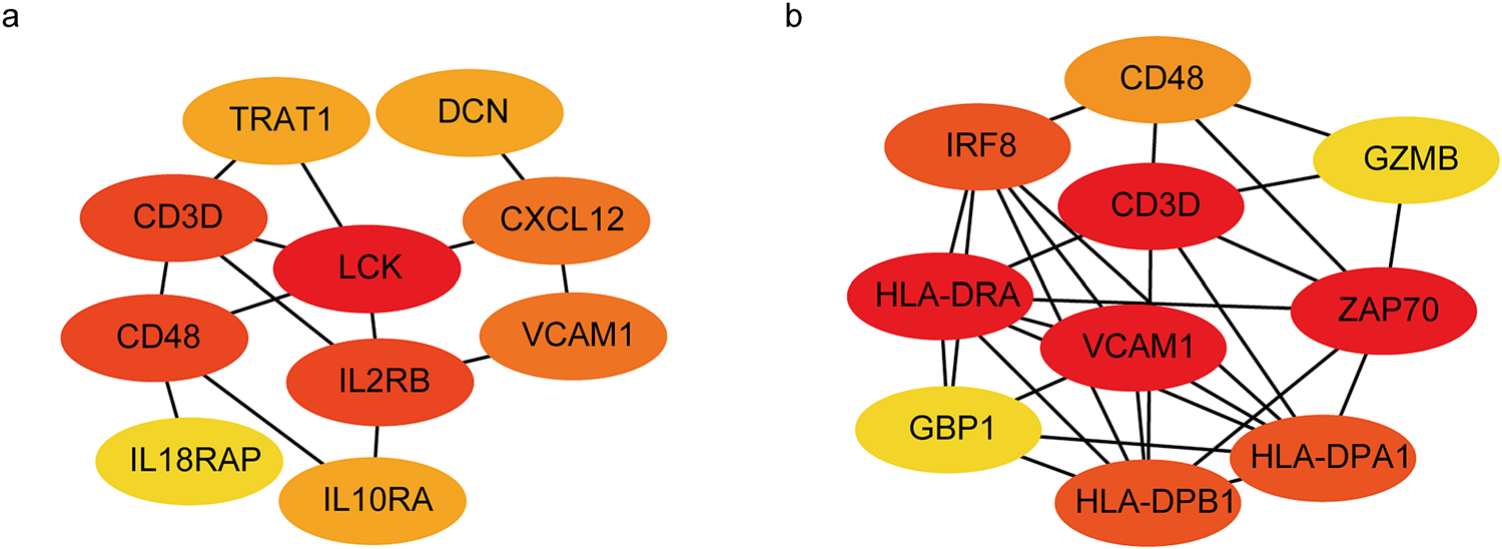
Results of degree algorithms from cytoHubba. (**a**) The PPI network of the top 10 hub genes associated with the FLT3-ITD mutation. (**b**) The PPI network of the top 10 hub genes associated with NPM1 mutations.

To further investigate the interaction among FLT3-ITD/NPM1-associated immune and stromal cell-relevant DEGs validated by GSE14468, we constructed PPI networks based on the STRING database and Cytoscape software. Then, we identified the top 10 FLT3-ITD/NPM1-associated hub genes by applying cytoHubba (Fig. 4). Node degree of importance is represented by circle color. As a result, the FLT3-ITD-associated hub genes were as follows: LCK, CD48, CD3D, IL2RB, CXCL12, VCAM1, DCN, IL10RA, TRAT1 and IL18RAP. NPM1-associated hub genes included VCAM1, CD3D, ZAP70, HLA-DRA, HLA-DPB1, HLA-DPA1, IRF8, CD48, GZMB and GBP1. LCK and VCAM1 were determined to be the most important hub genes in the networks associated with the FLT3-ITD mutation and the NPM1 mutation, respectively.

## Discussion

A number of genetic mutations have presented immune microenvironment modulatory properties in solid tumors: EGFR mutations correlate with an immunosuppressive TME and may impact the antitumor immune response in NSCLC^22,23^; TP53 and KRAS mutations in lung adenocarcinoma can regulate the immune microenvironment to affect PD-1 blockade immunotherapy^24,25^; JAK1 or JAK2 mutations may lead to acquired resistance to PD-1 blockade immunotherapy in patients with melanoma^26^. Recurrent genetic mutations found in AML have been heavily studied to classify and predict the risk of relapse after treatment. According to the ELN and NCCN guidelines, RUNX1, ASXL1, TP53, FLT3-ITD, NPM1 and biCEBPA mutations have been involved in AML prognostic stratification^2,3^. Interactions between leukemic stem cells and other cells in the BM microenvironment are known to be vital for the maintenance and progression of chemotherapy-resistant AML^14^. LSCs can remodel the BM niche into a favorable environment for expansion or even induce leukemic transformation. Nonetheless, the relationships between recurrent genetic mutations and the immune microenvironment in AML have not been comprehensively described^27^.

In our study, we calculated the immune and stromal scores for AML patients from the TCGA database based on the ESTIMATE algorithm. Our results showed that immune scores were significantly associated with OS and cytogenetic risk; a high immune score was a significantly poor prognostic factor for both the entire cohort and patients in the intermediate and poor cytogenetic risk groups, and a high stromal score tended to be correlated with poor OS in the intermediate and poor cytogenetic risk groups. The prognostic significance of immune and stromal scores was different in solid tumors: high immune and stromal scores correlated with poor survival in glioblastoma^18^, clear cell renal cell carcinoma (ccRCC)^28^ and gastric cancer^16^, whereas high immune and stromal scores correlated with better survival in cervical squamous cell carcinoma (CSCC)^29^ and pancreatic ductal adenocarcinoma (PDAC)^30^. These studies demonstrated the varied effects of immune and stromal scores on prognosis, and these effects are related to tumor type.

Due to the similar OS rate, immune scores and stromal scores between the intermediate and poor cytogenetic risk groups, we considered these patients as a single group to explore the characteristics of the somatic mutation-associated immune microenvironment in AML. We compared the immune/stromal scores, identified DEGs between the mutation and WT groups, conducted functional enrichment analysis of DEGs and selected somatic mutation-associated immune/stromal cell-relevant DEGs. We found that similar to the impact of immune and stromal scores on prognosis, distinct relationships existed between somatic mutations and immune/stromal scores. In other words, RUNX1, ASXL1 and TP53 mutations were related to higher immune or stromal scores, whereas FLT-ITD mutation was related to lower immune and stromal scores, although they were all poor prognostic mutations. Furthermore, patients with NPM1 mutation had lower stromal scores, while patients with biCEBPA mutation showed similar immune and stromal scores. despite their favorable prognostic risks.

The functional enrichment analysis and immune/stromal cell-relevant DEGs were generally consistent with the immune/stromal scores for individual genetic mutations. There are unique and common genes among mutations associated with immune/stromal cell-relevant DEGs. The results obtained in this study demonstrated that RUNX1, TP53 and ASXL1 mutation-associated characteristics of the microenvironment are similar. Reports have revealed the pro-inflammatory impact of RUNX1, TP53 and ASXL1 mutations on the immune microenvironment. RUNX1 mutation has been shown to activate NF-κB signaling and has been proposed to promote inflammatory signaling pathways in the bone marrow microenvironment^31^. Previous reports have shown that TP53 mutations induce pro-inflammatory effects on epithelial cells through NF-κB-mediated production of inflammatory cytokines. Moreover, TP53 mutations in CAFs are associated with pro-tumor and pro-inflammatory effects through enhanced production of cytokines and chemokines, including CXCL12, SDF-1 and IL-6, which notably affect the immune microenvironment^32,33^. ASXL1 mutation is one of the most frequently observed mutations leading to clonal hematopoiesis (CH), which have been known to show elevated inflammation, impaired tumor suppressor function, and risk of eventual hematological malignancy (HM)^34–36^. Patients with NPM1 mutation and FLT3-ITD mutation not only had similar lower scores but also had multiple common immune/stromal cell-relevant DEGs, which were not consistent with their opposite prognostic significance. Our results indicated that there might be a common mechanism on the impact of NPM1 and FLT3-ITD mutations on the bone marrow microenvironment, which remains to be explored.

Notably, several mutations associated with immune/stromal cell-relevant DEGs were observed to have prognostic significance in intermediate and poor cytogenetic risk patients. The results implied that these specific genes may play an important role in the formation of a mutation-associated microenvironment and may affect the survival of AML patients. Moreover, the majority of immune/stromal cell-relevant DEGs were confirmed to be significantly correlated with FLT3-ITD, NPM1, and biCEBPA mutations in the GSE14468 database. PPI networks were subsequently built based on the verified FLT3-ITD/NPM1-associated immune and stromal cell-relevant DEGs, and the top 10 hub genes were subsequently identified by the degree of interaction.

LCK (lymphocyte-specific protein tyrosine kinase) was the most significant hub gene associated with the FLT3-ITD mutation. LCK plays an essential role in the selection and maturation of developing T cells in the thymus, the activation of mature T-cells and the initiation of T cell antigen receptor (TCR) signal transduction pathways^37^. Studies have indicated higher expression of LCK in leukemic cells from less differentiated cases of AML (AML-0 and AML-1)^38^. A recent report found that LCK is overexpressed and mutated in CTV-1 cells (AML-M5 cell lines)^39^. Nonetheless, the expression of LCK in FLT3-ITD-mutated cells has not been studied to date. In the present study, the downregulation of LCK was correlated with the FLT3-ITD mutation. In a study of a zebrafish model^40^, FLT3 was found to initiate definitive hematopoietic stem cells, and the knockdown of FLT3 reduced hematopoiesis. The expression of the FLT3-ITD mutation resulted in the expansion of myeloid cells and the reduction of T cells. These results suggest that the FLT3-ITD mutation decreases the expression of LCK and reduces the production of functional T cells.

VCAM1 (vascular cell adhesion molecule-1) was shown to be the most significant hub gene associated with NPM1 mutation. VCAM1 is a cell adhesion molecule primarily expressed on endothelial cells, and its expression is induced by pro-inflammatory cytokines, such as TNFα^41,42^. VCAM1 has been identified to regulate vascular adhesion and transendothelial migration by binding to VLA-4 (very late antigen-4, an α4β1 integrin) on leukocytes. VCAM1 binding to VLA-4 confers AML blast cell protection from chemotherapy-induced apoptosis^43,44^. In our study, the downregulation of VCAM1 was confirmed to be correlated with NPM1 mutation. Although no study to date has explored the function of VCAM1 in NPM1-mutated AML patients, we speculated that the downregulation of VCAM1 may reduce the stroma-mediated protection of leukemic cells, which might confer favorable outcomes to AML patients with NPM1 mutations.

In conclusion, we focused on the relationship between recurrent genetic mutations and the immune microenvironment in AML patients based on TCGA database by integrated bioinformatic approaches. Important immune and stromal cell-relevant DEGs that affected the immune landscape of patients with individual gene mutations were identified and validated. Considering the specific properties of the hematopoietic microenvironment of leukemia^15^, ESTIMATE may not accurately predict infiltrating stromal and immune cells for the AML microenvironment, and we need to develop a more suitable and accurate algorithm. Due to the limited patient numbers in mutation subgroups in the TCGA database, further investigation of these mutation-associated stromal and immune signatures in large clinical AML patient cohorts is warranted, which may provide new prognostic biomarkers to achieve precision tumor therapy. Our results may help to elucidate how AML genetic mutations modulate the immune microenvironment to better guide personalized immunotherapy in the era of precision medicine^45^.

## Materials and methods

### Database

The transcriptional profiles and clinical and overall survival (OS) data of 173 AML patients were downloaded from the TCGA database (https://portal.gdc.cancer.gov/). The gene expression profile was measured experimentally using the Illumina HiSeq 2000 RNA Sequencing platform. Log2 transformations were performed for all gene expression data. Immune and stromal scores were calculated by applying the ESTIMATE algorithm^15^ to the mRNA expression data (https://bioinformatics.mdanderson.org/estimate/). The definitions of cytogenetic risk and risk-related somatic mutations were based on NCCN guidelines^2^.

For validation of the mutation-associated microenvironment signatures obtained from TCGA data, GSE14468 based on GPL570 (Affymetrix Human Genome U133 Plus 2.0 Array) were downloaded from the GEO database (https://www.ncbi.nlm.nih.gov/geo/), including cytogenetic risk, FLT3-ITD, NPM1 and CEBPA mutations.

### Identification of differentially expressed genes

AML patients in the intermediate and poor cytogenetics risk categories were divided into mutation and wild-type (WT) groups according to the individual somatic mutation status (RUNX1, TP53, ASXL1, FLT3-ITD, and NPM1). For CEBPA, patients with biallelic CEBPA mutation were classified as the biCEBPA group, and patients with monoallelic mutation or wild-type CEBPA were classified as the WT group. Differentially expressed genes (DEGs) were identified using the limma package in R software (version 3.6.2; https://www.r-project.org/). Genes with |log2FC|> 1.0 and adjusted *P* values (q values) < 0.05 were selected as DEGs. Volcano plots were generated using the ggplot2 package in R software.

### Functional enrichment analysis of DEGs

Functional enrichment analysis of DEGs was performed based on clusterProfiler, enrichplot, org.Hs.eg.db, and ggplot2 packages to identify the Gene Ontology (GO) categories, including biological processes (BP), cellular components (CC), and molecular functions (MF). Pathway enrichment analysis based on the Kyoto Encyclopedia of Genes and Genomes (KEGG) database^46,47^ was also conducted using these packages. Upregulated and downregulated DEGs were annotated by functional enrichment analyses, and FDR (false discovery rate) < 0.05 was considered to be significant. The top 10 GO terms in each of the BP, CC, MF and top 30 KEGG pathways are presented using bar plots.

### Immune/stromal cell-relevant DEGs and overall survival analysis

According to genes selected by the ESTIMATE algorithm, immune/stromal cell-relevant DEGs of each mutation group were identified. To explore the prognostic value of these immune/stromal cell-relevant DEGs in predicting the overall survival of AML patients in the intermediate and poor cytogenetic risk groups, Kaplan–Meier survival curves were generated by the "survival” package in R software using the log-rank test. *P* values < 0.05 were considered to be significant.

### Protein–protein interaction (PPI) network and hub genes

Protein–protein interaction (PPI) network construction of FLT3-ITD/NPM1-associated immune and stromal cell-relevant DEGs validated by GSE14468 was based on the STRING online database (version 11.0; https://string-db.org/) and Cytoscape software (version 3.6.0; https://cytoscape.org/). We used cytoHubba to identify the top 10 hub genes according to the degree algorithm.

### Statistical analysis

Comparisons of immune and stromal scores among cytogenetic risk groups were performed using one-way analysis of variance. Comparisons of immune and stromal scores between the mutation and WT groups were performed using the Mann–Whitney U test. Survival functions were estimated using the Kaplan–Meier method and were compared using the log-rank test. The SPSS Statistics 22.0 (IBM Corp. in Armonk, NY; https://www.ibm.com/) and GraphPad Prism 5.0 (GraphPad Software, La Jolla California USA, www.graphpad.com) were used for the data analysis.

## Supplementary information

Supplementary Information.

## References

[1] Ferrara F, Schiffer CA (2013). Acute myeloid leukaemia in adults. Lancet.

[2] Tallman MS (2019). Acute myeloid leukemia, version 3.2019, NCCN clinical practice guidelines in oncology. J. Natl. Compr. Cancer Netw. JNCCN.

[3] Döhner H (2017). Diagnosis and management of AML in adults: 2017 ELN recommendations from an international expert panel. Blood.

[4] Hanahan D, Weinberg RA (2011). Hallmarks of cancer: the next generation. Cell.

[5] Hanahan D, Coussens LM (2012). Accessories to the crime: functions of cells recruited to the tumor microenvironment. Cancer Cell.

[6] Pitt JM (2016). Targeting the tumor microenvironment: removing obstruction to anticancer immune responses and immunotherapy. Ann. Oncol..

[7] Wu T, Dai Y (2017). Tumor microenvironment and therapeutic response. Cancer Lett..

[8] Achyut BR, Arbab AS (2016). Myeloid cell signatures in tumor microenvironment predicts therapeutic response in cancer. OncoTargets and therapy.

[9] Gbolahan OB (2017). Immunotherapeutic concepts to target acute myeloid leukemia: focusing on the role of monoclonal antibodies, hypomethylating agents and the leukemic microenvironment. Int. J. Mol. Sci..

[10] Binnewies M (2018). Understanding the tumor immune microenvironment (TIME) for effective therapy. Nat. Med..

[11] Lamble AJ, Lind EF (2018). Targeting the immune microenvironment in acute myeloid leukemia: a focus on t cell immunity. Front. Oncol..

[12] Shafi AA (2018). Patient-derived models reveal impact of the tumor microenvironment on therapeutic response. Eur. Urol. Oncol..

[13] Taube JM (2018). Implications of the tumor immune microenvironment for staging and therapeutics. Mod. Pathol..

[14] Ladikou EE, Sivaloganathan H, Pepper A, Chevassut T (2020). Acute myeloid leukaemia in its niche: the bone marrow microenvironment in acute myeloid leukaemia. Curr. Oncol. Rep..

[15] Yoshihara K (2013). Inferring tumour purity and stromal and immune cell admixture from expression data. Nat. Commun..

[16] Wang H, Wu X, Chen Y (2019). Stromal-immune score-based gene signature: a prognosis stratification tool in gastric cancer. Front. Oncol..

[17] Xie P (2019). Development of an immune-related prognostic signature in breast cancer. Front. Genet..

[18] Jia D (2018). Mining TCGA database for genes of prognostic value in glioblastoma microenvironment. Aging.

[19] Huang S (2019). Identification of prognostic genes in the acute myeloid leukemia microenvironment. Aging.

[20] Ni J (2019). Screening the cancer genome atlas database for genes of prognostic value in acute myeloid leukemia. Front. Oncol..

[21] Yan H (2019). Identification of prognostic genes in the acute myeloid leukemia immune microenvironment based on TCGA data analysis. Cancer Immunol. Immunother. CII.

[22] Lin A, Wei T, Meng H, Luo P, Zhang J (2019). Role of the dynamic tumor microenvironment in controversies regarding immune checkpoint inhibitors for the treatment of non-small cell lung cancer (NSCLC) with EGFR mutations. Mol. Cancer.

[23] Jia Y (2019). EGFR-targeted therapy alters the tumor microenvironment in EGFR-driven lung tumors: Implications for combination therapies. Int. J. Cancer.

[24] Biton J (2018). TP53, STK11, and EGFR mutations predict tumor immune profile and the response to anti-PD-1 in lung adenocarcinoma. Clin. Cancer Res..

[25] Dong ZY (2017). Potential predictive value of TP53 and KRAS mutation status for response to PD-1 blockade immunotherapy in lung adenocarcinoma. Clin. Cancer Res..

[26] Zaretsky JM (2016). Mutations associated with acquired resistance to PD-1 blockade in melanoma. N. Engl. J. Med..

[27] Mendez LM, Posey RR, Pandolfi PP (2019). The interplay between the genetic and immune landscapes of aml: mechanisms and implications for risk stratification and therapy. Front. Oncol..

[28] Xu WH (2019). Prognostic value and immune infiltration of novel signatures in clear cell renal cell carcinoma microenvironment. Aging.

[29] Pan XB, Lu Y, Huang JL, Long Y, Yao DS (2019). Prognostic genes in the tumor microenvironment in cervical squamous cell carcinoma. Aging.

[30] Pu N (2019). Genetic landscape of prognostic value in pancreatic ductal adenocarcinoma microenvironment. Ann. Transl. Med..

[31] Nakagawa M (2011). AML1/RUNX1 functions as a cytoplasmic attenuator of NF-κB signaling in the repression of myeloid tumors. Blood.

[32] Taura M (2008). p53 regulates Toll-like receptor 3 expression and function in human epithelial cell lines. Mol. Cell. Biol..

[33] Cui Y, Guo G (2016). Immunomodulatory function of the tumor suppressor p53 in host immune response and the tumor microenvironment. Int. J. Mol. Sci..

[34] Genovese G (2014). Clonal hematopoiesis and blood-cancer risk inferred from blood DNA sequence. N. Engl. J. Med..

[35] Jaiswal S (2014). Age-related clonal hematopoiesis associated with adverse outcomes. N. Engl. J. Med..

[36] Kaner J (2020). Clonal hematopoiesis and premalignant diseases. Cold Spring Harbor Perspect. Med..

[37] Alarcón B, van Santen HM (2010). Two receptors, two kinases, and T cell lineage determination. Sci. Signal..

[38] Rouer E, Dreyfus F, Melle J, Benarous R (1994). Pattern of expression of five alternative transcripts of the lck gene in different hematopoietic malignancies: correlation of the level of lck messenger RNA I B with the immature phenotype of the malignancy. Cell Growth Differ.

[39] Li L, Cui Y, Shen J, Dobson H, Sun G (2019). Evidence for activated Lck protein tyrosine kinase as the driver of proliferation in acute myeloid leukemia cell, CTV-1. Leuk. Res..

[40] He BL (2014). Functions of flt3 in zebrafish hematopoiesis and its relevance to human acute myeloid leukemia. Blood.

[41] Osborn L (1989). Direct expression cloning of vascular cell adhesion molecule 1, a cytokine-induced endothelial protein that binds to lymphocytes. Cell.

[42] Kong DH, Kim YK, Kim MR, Jang JH, Lee S (2018). Emerging roles of vascular cell adhesion molecule-1 (VCAM-1) in immunological disorders and cancer. Int. J. Mol. Sci..

[43] Cavenagh JD, Gordon-Smith EC, Gibson FM, Gordon MY (1993). Acute myeloid leukaemia blast cells bind to human endothelium in vitro utilizing E-selectin and vascular cell adhesion molecule-1 (VCAM-1). Br. J. Haematol..

[44] Lévesque JP, Helwani FM, Winkler IG (2010). The endosteal 'osteoblastic' niche and its role in hematopoietic stem cell homing and mobilization. Leukemia.

[45] Scheetz L (2019). Engineering patient-specific cancer immunotherapies. Nat. Biomed. Eng..

[46] Kanehisa M, Goto S (2000). KEGG: Kyoto encyclopedia of genes and genomes. Nucleic Acids Res..

[47] Kanehisa M, Sato Y, Furumichi M, Morishima K, Tanabe M (2018). New approach for understanding genome variations in KEGG. Nucleic Acids Res..

