# Supplementary material for: Characterization of somatic mutation-associated microenvironment signatures in acute myeloid leukemia patients based on TCGA analysis
Source: Sci Rep. 2020 Nov 4;10:19037. doi: 10.1038/s41598-020-76048-8 (PMC7643165; doi:10.1038/s41598-020-76048-8)
Supplement: Supplementary file 1 — Supplementary Information. [file 41598_2020_76048_MOESM1_ESM.pdf]

Characterization of somatic mutation associated microenvironment in acute myeloid leukemia patients based on TCGA analysis

Authors

Jun Wang, Feng-Ting Dao, Lu Yang, Ya-Zhen Qin*

Supplementary Figure 1

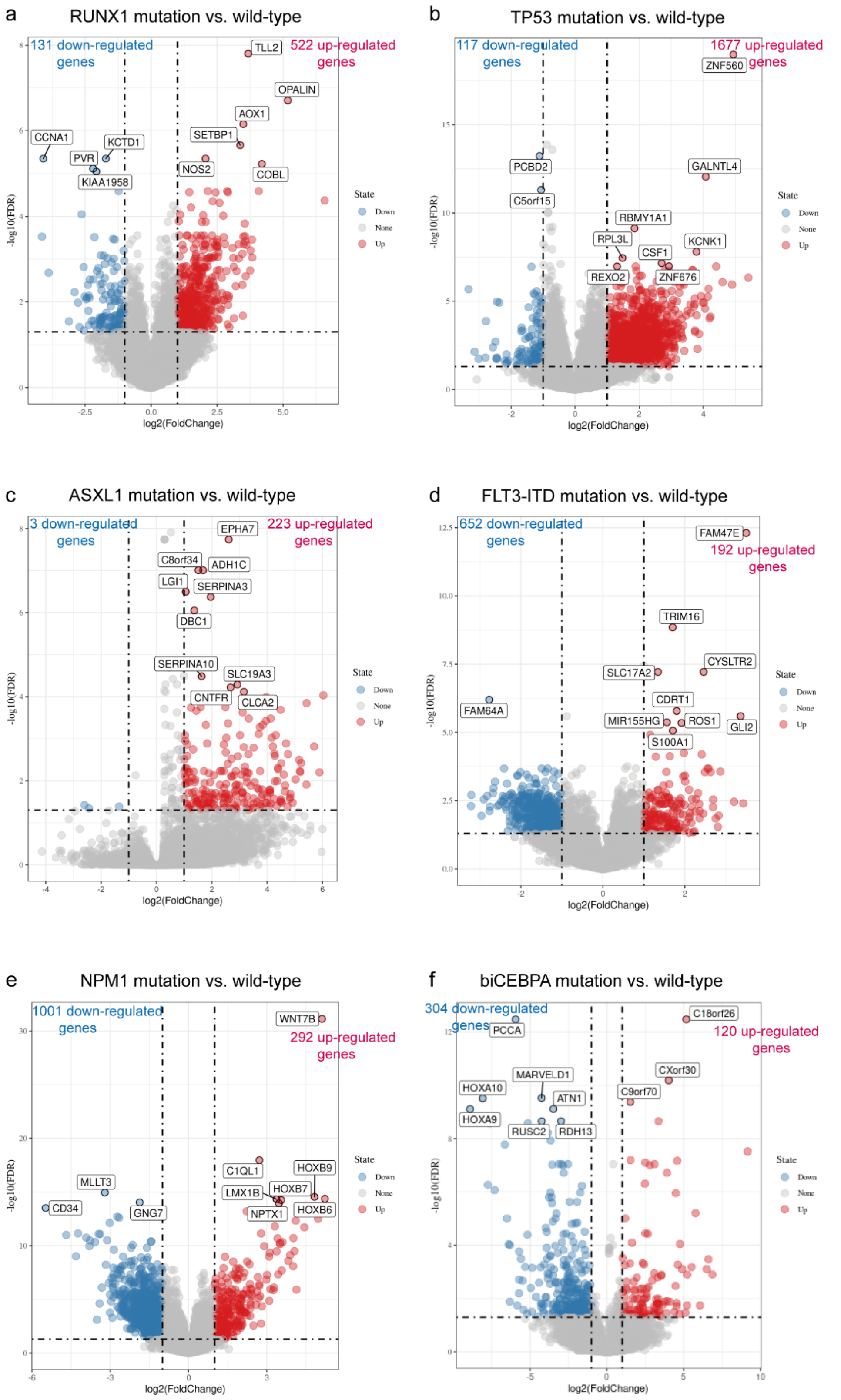

Supplementary Figure 1 Identification of DEGs based on mutations **a-f.** Volcano plots of DEGs of mutation vs. WT (|log2FC|>1.0, q-value < 0.05).

Supplementary Figure 2

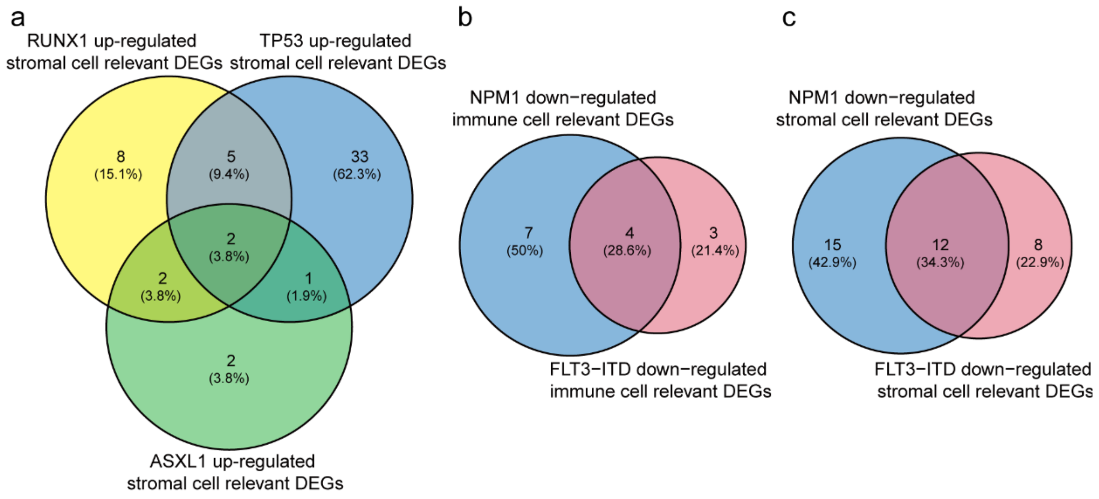

Supplementary Figure 2 Commonly immune and stromal cells relevant DEGs associated with mutations Venn plots were performed to reveal (**a**) intersect up-regulated stromal cell relevant DEGs among RUNX1, TP53 and ASXL1 mutations (**b**) intersect down-regulated immune cell relevant DEGs between FLT3-ITD and NPM1 mutations (**c**) intersect down-regulated stromal cell relevant DEGs between FLT3-ITD and NPM1 mutations.

Supplementary Figure 3

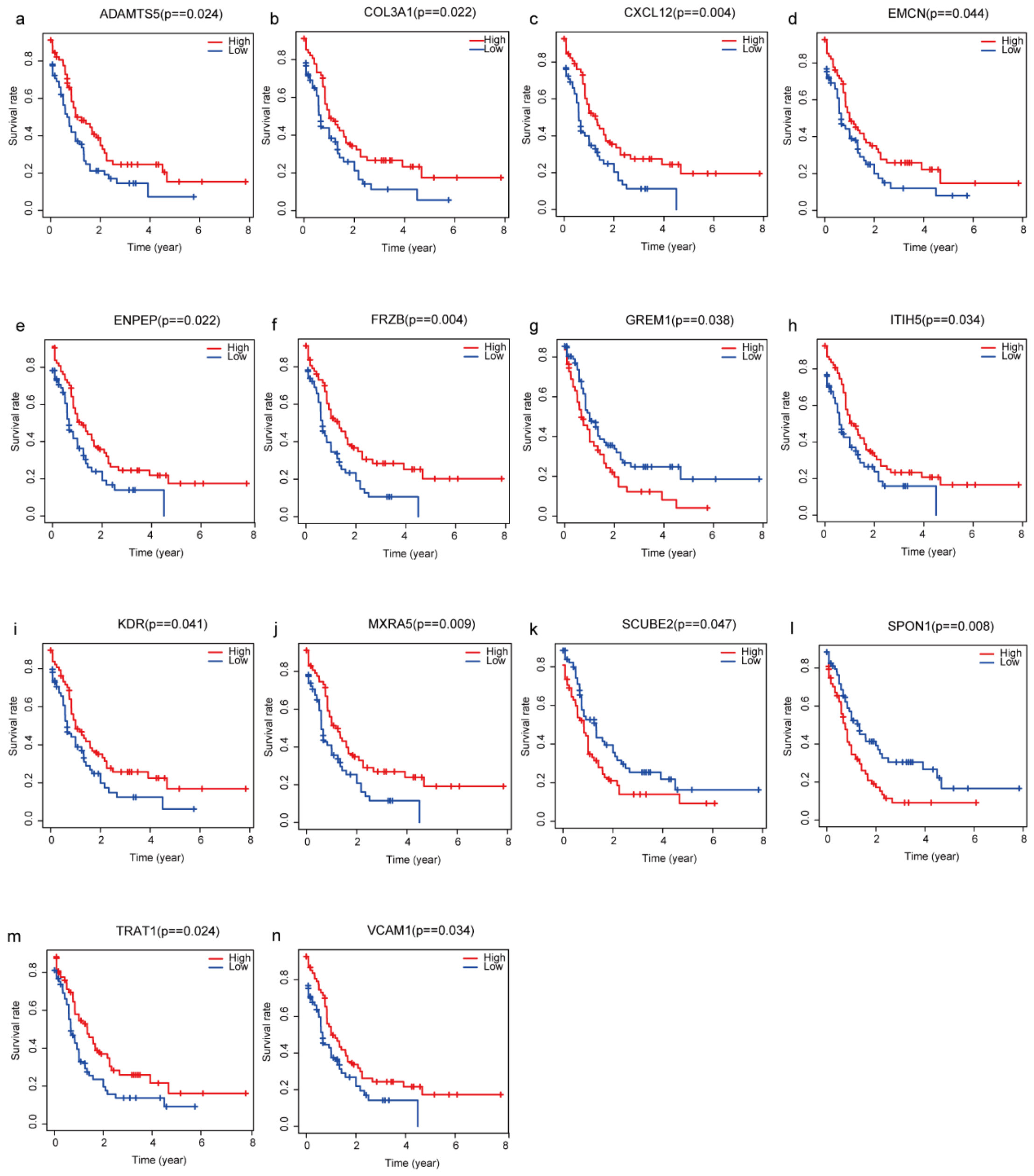

Supplementary Figure 3 The prognostic significance of mutation associated immune and stromal cells relevant DEGs in the AML intermediate- and poor-cytogenetic risk patients Kaplan-Meier survival analysis for signatures following comparison of high vs. low gene expression groups according to the median value of each gene (log-rank test, $p < 0.05$).

Supplementary Table 1 The mutation associated immune/stromal cells relevant DEGs

		Immune cells relevant DEGs	Stromal cells relevant DEGs
RUNX1 mutation	up-regulated	CD74; HLA-DMA; HLA-DPA1; HLA-DPB1; HLA-DRA; IL10RA; IRF8; SELL（n=8）	ABCA6; CD200; COL8A2; CXCL14; DDR2; FRZB; HGF; ITIH5; PAPPA; PDE2A; PDGFRB; PLXNC; TLR7; RARRES2; SCUBE2; SGCD; SULF1 (n=17)
	down-regulated	No	ATP8B4; C3AR1 (n=2)
TP53 mutation	up-regulated	CCR7; CD2; CD247; CD27; CD3D; CST7; GBP1; GNLY; GZMB; GZMH; GZMK; IL2RB; IL32; IL18RAP; IL7R; ITK; KLRB1; KLRK1; LCK; LTB; PRF1; RGS1; RHOH; ZAP70 (n=24)	AOC3; BGN; C1QB; CD248; CDH5; CILP; COL14A1; COL15A1; COL1A2; COL3A1; COL5A3; COL6A3; CXCL14; CXCL9; DDR2; ENPEP; FASLG; FBLN2; FRZB; GIMAP5; HEPH; IGF1; IL18R1; IL1B; ISLR; ITGBL1; KCNJ8; KDR; LRRC32; MAF; MXRA5; PAPPA; PDE2A; PLXDC1; PTGER3; RAMP3; RUNX1T1; SGCD; SPON1; SULF1; VCAM1 (n=41)
	down-regulated	EVI2B; LAIR1 (n=2)	No
ASXL1 mutation	up-regulated	No	DDR2; FRZB; GREM1; ISLR; ITIH5; PRKG1; RARRES2 (n=7)
	down-regulated	No	ATP8B4 (n=1)
FLT3-ITD mutation	up-regulated	No	ENPP2; PTGIS (n=2)
	down-regulated	CD3D; CD48; GBP1; IL10RA; IL18RAP; IL2RB; LCK (n=7)	AOC3; BGN; C1QB; CDH5; COL15A1; COL1A2; COL3A1; COL6A3; CXCL12; DCN; EMCN; FRZB; ISLR; ITIH5; KDR; MXRA5; PDE2A; RARRES2; TRAT1; VCAM1 (n=20)

NPM1 mutation	up-regulated	CSTA; SRGN (n=2)	ADAMTS5; C3AR1; F13A1; TNN; RASGRP3 (n=5)
	down-regulated	CD3D; CD48; GBP1; GZMB; HLA-DPA1; HLA-DPB1; IRF8; HLA-DRA; IL18RAP; RGS1; ZAP70 (n=11)	BGN; CD200; CD248; CDH5; COL1A2; COL6A3; COL8A2; CXCL12; DCN; EDIL3; ENPEP; ERG; FBLN2; FRZB; HGF; IL18R1; ISLR; ITIH5; ITM2A; MXRA5; PAPPA; PRKG1; SIGLEC1; SPON1; SULF1; TRAT1; VCAM1 (n=27)
biCEBPA mutation	up-regulated	IL2RG (n=1)	ITM2A (n=1)
	down-regulated	GBP2; IL4R (n=2)	F13A1 (n=1)
